# Responding to the Public during a Pandemic: Perceptions of ‘Satisfactory’ and ‘Unsatisfactory’ Policing

**DOI:** 10.1093/police/paab058

**Published:** 2021-09-06

**Authors:** Aram Ghaemmaghami, Rob Inkpen, Sarah Charman, Camille Ilett, Stephanie Bennett, Paul Smith, Geoff Newiss

**Affiliations:** 1 Aram Ghaemmaghami, Lecturer in Policing and Criminal Justice, School of Criminology and Criminal Justice, University of Portsmouth, Portsmouth, UK; 2 Rob Inkpen, Reader in Physical Geography, School of the Environment, Geography and Geosciences, University of Portsmouth, Portsmouth, UK; 3 Sarah Charman, Professor of Criminology, School of Criminology and Criminal Justice, University of Portsmouth, Portsmouth, UK. E-mail: sarah.charman@port.ac.uk; 4 Camille Ilett, Research Associate, School of Criminology and Criminal Justice, University of Portsmouth, Portsmouth, UK; 5 Stephanie Bennett, Lecturer in Criminology and Forensic Psychology, University of Chichester, UK; 6 Paul Smith, Reader in Crime Science, School of Criminology and Criminal Justice, University of Portsmouth, Portsmouth, UK; 7 Geoff Newiss, Research Associate, School of Criminology and Criminal Justice, University of Portsmouth, Portsmouth, UK

## Abstract

As part of a substantial research project on policing the Covid-19 pandemic, a public survey was conducted in Hampshire and the Isle of Wight in England. Four open-ended questions provided participants with the opportunity to produce unlimited free-text responses regarding their perception of policing during the pandemic. Responses were coded and thematically analysed to identify themes concerning public compliance and policing during the lockdown. Subthemes surrounding communication, efficiency, and equity emerged from participant’s perceptions of what they considered to be ‘satisfactory’ and ‘unsatisfactory’ forms of policing during the pandemic. A common sub-theme regarding the public’s confusion over the role of the police was countered by an acknowledgement that the police were ‘doing their best.’ The pandemic has thrown into sharper relief pre-crisis public perceptions of appropriate policing. The free-text responses highlight the ongoing tensions between normative and instrumental approaches to policing and public expectations of police actions.

## Introduction

On 3 March 2020, the UK government put in place a plan ‘to contain, delay, research, and mitigate’ the spread of a new form of a highly infectious coronavirus—Covid-19—that was sweeping its way across the world at an unprecedented rate. The initial plan to contain the virus had little success and on 23 March 2020, Prime Minister Boris Johnson imposed a ‘stay at home’ order, imploring people during a live televised address ‘to stay at home, protect our NHS, and save lives’ (HM Government, 2020a). During the months that followed, several lockdown restrictions were put in place, severely limiting civil liberties. The implementation of lockdown restrictions across the UK has been further complicated by the powers afforded to the devolved nations by the Coronavirus Act 2020, with Wales, Scotland, and Northern Ireland all choosing to pursue different strategies from the UK Parliament at various stages during the pandemic (Paun *et al.*, 2020). Those charged with managing compliance with these restrictions—the police—have been put under increased public pressure and scrutiny from the inception of the restrictions.

‘Policing by consent’ is generally considered to be central to the UK policing model but may be potentially under threat by the changes to police powers following the emergence of Covid-19 (Bradford *et al.*, 2020). For instance, under sub-section 52 of the Coronavirus Act 2020, the police have been given extended powers under section 22 public order offences to restrict the movement of individuals and even detain those who do not cooperate—using reasonable force where necessary (HM Government, 2020b). The powers to restrict the free-movement of individuals in public spaces would be almost unthinkable in almost any other context. However, the nature of the virus and its dysphoric effect on the public’s levels of ‘threat perception’ has created many unique challenges for policing services worldwide (Clark *et al.*, 2020, p. 77). The police have been put in the unenviable position of balancing the needs of a population with a dichotomy of opinion: some believing the pandemic specific regulations to be an overextension of policing powers and a ‘threat to freedom’, with others believing them to be essential in order to ‘beat the virus’ (Grace, 2021, p. 1036). In short, the pandemic has made it very challenging for the police to maintain ‘public trust and confidence’ across large sections of the population—which is central to their ability to maintain legitimacy (Jones, 2020, p. 580).

Both gaining and maintaining the trust of the public is paramount to obtaining a level of ‘voluntary’ or ‘normative’ compliance (Tyler, 1990; Bargain and Aminjonov, 2020). The unprecedented nature of the pandemic and its effects on the social, economic, and political landscape has created a need for research that examines police–public relations during the Covid-19 crisis. Research across these contexts has been rapidly emerging to increase our understanding of police–public relations and their effects on compliance with lockdown restrictions, not just within a national UK context, but on a global scale. This study aims to add to this discourse—to provide critical oversight over the emerging trends in public–police relations within the paradigm the pandemic presents. It does so by analysing the responses to the open-ended questions within a public survey conducted in the policing areas of Hampshire and the Isle of Wight, during the period between July and September 2020. The contents of these free-text responses are narrative rich, and have allowed this study to examine public perceptions of what is considered ‘satisfactory’ and ‘unsatisfactory’ policing during the pandemic. It also considers whether these perceptions of policing are an amplification of ‘normal’ public expectations of policing, or whether they are specific to the Covid-19 response. In the following section, the wider context of police–public engagement is examined in more detail—specially the nature of policing by consent and how this is affected in times of crisis.

## Context: the realities of adopting the policing by consent model during the pandemic

Striking a balance between public perceptions of what constitutes a ‘fair’ application of policing powers and demonstrably effective methods of policing are a process that habitually produces diminishing returns for those charged with navigating public–police relations. More often than not, the police have to reach a compromise with their public adjudicators—to adopt approaches that are ‘good enough’, where they both fulfil their responsibility to prevent and manage criminal behaviour and to promote practice that the public consider to be ‘fair and efficient’ (Bowling, 2007, p. 22). In times of national crisis—such as that presented by the Covid-19 pandemic—public consensus on what exactly constitutes fair policing is subject to constant fluctuation (Hoffman, 2019). The pandemic has necessitated the production of policy designed ‘to coerce citizens’ into compliance with liberty depriving restrictions. Such policy can only be successfully policed if the response is perceived to be ‘proportionate’ to the level of threat (Bowling *et al.*, 2017).

Evidence suggests that the acceptance of the use of ‘intrusive powers’ by the police during the pandemic is dependent on levels of confidence in the police to use such powers responsibly (Yesberg *et al.*, 2021, p. 11). The nature of the pandemic, where the threat level is in a constant state of flux, presents a substantial challenge to the police—both in terms of compliance with restrictions and in ensuring general public order. This challenge is further compounded by the transient nature of public discourse during the pandemic. For example, the outcome of key events, such as the May 2020 media story regarding Dominic Cummings (then chief advisor to the Prime Minister) seemingly breaking lockdown restrictions, can influence public consensus on the legitimacy of the use of police powers. However, such fluctuations in public sentiment are often ‘short lived’; gaining a more nuanced understanding of the factors driving the ‘emotional response’ of the public is a more reliable predictor of public–police relations in the longer term (Yesberg *et al.*, 2021, pp. 11–12).

The development of policing practice has traditionally been curtailed by the police’s ability to circumnavigate the field of ‘ritualistic behavioural norms’ found within wider society—to meet public expectations and standards, while also ensuring public order and safety, which rarely coalesce (McCarthy, 2014, p. 159). The pandemic has dramatically changed the normative language of society, where the balance between the ‘injunctive norms’ put upon the individual—which in terms of policing equates to the moral imperative to follow the rule of law—have exacerbated the public consensus on who is described by the ‘descriptive norm’, in this context, those that are perceived to break the law. It is important to note that the construction of ‘injunctive norms’ cannot be made in isolation of ‘descriptive norms’ (Livingstone, 2020, pp. 10–11). In effect, a consensus on the identity of those who break the law is essential for defining the boundaries of the identity of those who do comply with the law: to know oneself is to know one’s enemy. Where there is ambiguity, or where no obvious ‘descriptive norm’ exists, such a norm will be created (Lois and Wessa, 2021).

Whether this norm is based in reality or not is immaterial, the effect of the norm being practiced by the public creates real world, material effects. In the context of the Covid-19 pandemic, the perceived prevalence of those viewed to be breaking specific restrictions has the potential to impact on what is considered to be a ‘fair’ use of police powers, as the metrics of criminality have significantly shifted as a result of the pandemic (Reicher and Stott, 2020, p. 696). Rising levels of ‘fear of crime’ within a population have long been known to ‘decrease collective trust and cohesion’ in the police (Jackson and Gray, 2010, pp. 1–2). Equally, feelings of ‘victimization’ will increase the levels of ‘dysfunctional fear’ at the individual and group level, which can lead to anxieties clouding judgment on what is considered an acceptable use of police powers (Lee *et al.*, 2020, p. 331).

In a very real sense, everybody in society has become a victim of the pandemic, whether that is through the loss of their freedom of movement, through the loss of employment, through the loss of contact, or in the worst cases, through the loss of loved ones. In order to understand the dynamics driving public–police relations during the pandemic, such considerations must be taken into account. Equally, the police have faced a significant challenge during the course of the pandemic in terms of reinforcing behavioural norms, with several authorities vying for influence over what such norms should look like in real terms. Although the legal apparatus that informed their practice was institutional—with the government setting lockdown restrictions through legislation—the implementation of such restrictions was constantly being informed by a wide range of ‘experts’ from both the civic and public domain, including (but not limited to) scientists, public health professionals, and the media. As such, it is difficult to pinpoint exactly where public perceptions of policing are based on evidence of practice, or where they are informed by the media hyperbole that surrounds the discourse of Covid-19 (Solymosi *et al.*, 2020). Although this study does not proclaim to categorically measure the antecedents of public–police discourse during the pandemic, its findings do elucidate the nature of public opinion regarding policing during the initial lockdown phase. In the following section—research rationale—the precise metrics of this study are explained in more detail.

## Research rationale

The study of free-text responses—open-ended question answers—from an online public survey conducted across the policing area of Hampshire and the Isle of Wight in the south of England are the basis of this study’s qualitative enquiry. This study is part of a wider joint ESRC funded research project between the University of Portsmouth and Hampshire Constabulary, which has been designed to examine the impact of pandemic policing on both the police and the public. During the period following the initial lockdown period, an online survey was distributed between 28 July and 15 September 2020 using the JISC Online Survey platform. The survey was initially advertised through Hampshire Constabulary and their community networks as well as Facebook pages of local police stations. In addition, a dedicated Facebook page was set up and linked to the survey to ensure that it was not necessarily viewed as directly attached to the police force. University press releases to local media across Hampshire and the Isle of Wight as well individual contact with community groups within the area was undertaken to try to cover as diverse a population as possible. It is accepted that there may be a bias in sampling to those groups that use the Internet and have access to Facebook. However, the distribution of ages implies that the sampling was not biased towards a younger demographic.

Similarly, it is recognized that asking about self-compliance is likely to produce some dishonest responses as individuals may be unwilling to suggest they have broken the law, and likewise for encounters with the police. The data, particularly for key workers, implied that individuals were willing to identify their own deviations from lockdown regulations to some extent. As the key concern for the survey was public perception, it was felt that these potential sources of error also represented individual’s perception of their own law-abiding status. Questions on the compliance of others were likely to be more informative as there was less reason for individuals to be concerned about presenting such a law-abiding image of others.

As Table  1 illustrates, the survey was not necessarily representative of the population of the region in terms of the distribution of age and gender. The ethnic diversity of the survey is harder to gauge. Although the key figures in the survey are similar to those for the region as whole, with 94.9% of the survey being ‘White British’ or ‘White Other’ (the region as whole was 95% of the population in these classes), 3.1% of the respondents in the survey preferred not to give their ethnicity. Table  1 illustrates that the survey was over-represented in females in the middle and latter age groups relative to the population of the region as a whole. It also highlights the percentage contribution to the free-text comments as 60% of respondents overall (although 12 of these stated they preferred not to provide either their gender or age group so have been excluded from the analysis below). A similar distribution of comments within the female, middle to later age groups can also be seen, much in keeping with the distribution of the population within the survey itself. This means that the survey is more reflective of this particular section of the population than the population of the region as a whole, but still contains useful information on the perceptions of policing within the pandemic, but with the caution that the comments and themes need to be viewed in the light of the nature of the population contributing the comments.

**Table 1:** Percentage in each age/gender group providing information

	Region		Survey		Free-text	
Age group (years)	Male	Female	Male	Female	Male	Female
18–24	4.5	4.1	1.1	1.8	0.4	2.2
25–34	6.8	6.9	2.3	7.2	2.0	7.0
35–44	7.1	7.6	5.4	10.4	6.3	8.7
45–54	8.7	9.3	7.0	19.2	7.4	19.3
55–64	8.3	8.6	8.4	16.2	8.7	15.0
65–74	7.1	7.7	9.7	8.1	11.4	7.8
75+	5.7	7.5	2.3	1.1	2.7	0.9
Number of responses	Not applicable	Not applicable	270	477	174	273

The survey itself had 36 questions. It began with questions about the individual’s personal information such as gender, ethnicity, and income. The next section concerned the individual’s perception of policing and was structured around the existing surveys carried out by Hampshire Constabulary. This information supplemented their existing data on public perceptions. The next section concerned issues of compliance, while the final section contained questions designed to undercover more about the worldviews of individuals. This paper is focused on the free-text comments that individuals made at the end of the policing question section. At this point, the individuals would be expected to be engaged with the survey and thinking about policing in general and within their local area. It was hoped that inserting free-text questions at this juncture would provide a more discursive outlet for issues identified by the individual when answering the previous questions in the survey. The four questions relate to:

1. Whether an individual had contact with the police during lockdown and to briefly ascertain its nature (i.e. direct contact, witnessing police action, or in passing).
2. A description of this encounter in more detail.
3. To ascertain whether the outcome of this contact was satisfactory or not (and why).
4. Opinions on the policing of lockdown restrictions in their local area in more general terms.

This study is designed to contribute to the overall understanding of the dynamic between the police and the public by constructing a picture of how the public perceive policing in the pandemic. It also highlights the need for a coherent and consistent image of public perceptions of what is ‘satisfactory’ and ‘unsatisfactory’ policing during the pandemic and how far this deviates from the public’s perception of ‘normal’ policing—that is to say the role the police have played within the community setting prior to the advent of Covid-19 restrictions. Engendering such an understanding is paramount if the police are to maintain manageable levels of public confidence in their ability to perform their duties with efficacy; maintaining trust in the police is crucial to ensuring compliance, both during the pandemic and in any subsequent events that may follow it (Murphy, 2017).

The free-text responses underwent a form of content analysis, details of which can be found in the following section, along with a breakdown of the individual research questions used by this study and the rationale for their implementation.

## Methodology

The free-text responses in the survey allow the contextualization of the factors that drive levels of compliance, as well as overall police satisfaction. It also provides an opportunity to speculate upon public reasoning that pertains to Covid-19 restrictions specifically, and that of public–police relations in more general terms. With that rationale in mind, the analysis of the free-text data derived from the survey has been designed to address two specific research questions:

1. How do people perceive ‘satisfactory’ and ‘unsatisfactory’ policing of the pandemic?
2. Are these perceptions a magnification of public expectations of policing outside of the Covid-19 restrictions, or are they pandemic specific?

The free-text comments were initially coded for their content using NVivo 12, and were then thematically analysed to elicit the possible relationships between them.

This study has employed a content-analysis approach in order to explore this data—to identify any emergent themes and to speculate on their explanatory value in relation to our understanding of compliance and how that relates to the policing of lockdown restrictions. It has also allowed for further discussion on the relationship between public–police relations in more general terms; to deliberate on the extent to which general public–police relations apply within the context of the Covid-19 pandemic and it’s lockdown measures.

In short, content analysis has been employed to bridge the gap between the quantitative nature of the survey and the qualitative aims of this study: to analyse the free-text responses (Schreier *et al.*, 2019, p. 5). It has allowed for each response to be coded in accordance with emergent themes—to produce a map of common attitudes and their interconnections with one another, while also exploring the hierarchy of priorities within the collective conciseness of respondents (Schreier *et al.*, 2020, p. 11). Before content analysis could take place, the units of analysis had to be clearly defined. In some respects, this task has been straightforward—the units of analysis are confined within the open-ended questions presented within the survey. The focus of these questions, which relate to the public’s experiences of policing during lockdown—be they a direct interaction, through observation at the street level, or through media representation—was the initial starting point for the thematic analysis of the data. It quickly became apparent that the data were more convoluted than this. The majority of respondents were reporting on their perceptions based on both their personal observation and media representation, with little distinction made between the two.

The use of qualitative data analysis software (QDAS)—NVivo 12—has allowed for this relatively large population of qualitative data to be processed while producing outputs that visually represent both the themes within the dataset and how they connect. Where it has specifically aided this study is in its ability to aggregate the qualitative data from the quantitative format of the survey. In particular, it has allowed for the contextualization of the responses—taking into account the wider survey and how its format may have impacted on the content of the free-text responses.

The starting point of any form of thematic analysis must begin by relating the data obtained throughout the study to the problematic that instigated its manufacture (Seal, 2016). The survey structure guided the initial scoping of the free-text data, with the emergent themes developing from that exercise. The nature of how the data have been offered by respondents has also been taken into account during the analysis; free-text responses by their very nature have been offered up freely. Although the structure of the survey constrains the type of response each participant can make, a ‘subjective’ approach to the analysis of the content of responses must be adopted if their true explanatory value is to be uncovered (Bishop, 2017, p. 4). The content analysis of the responses began with the scoping of the ‘narrative functions’ being used by respondents to express their views; separating the attitudes that led to the statement being made from the specific contents of each statement (Franzosi, 2010, p. 4).

**Figure 1:**
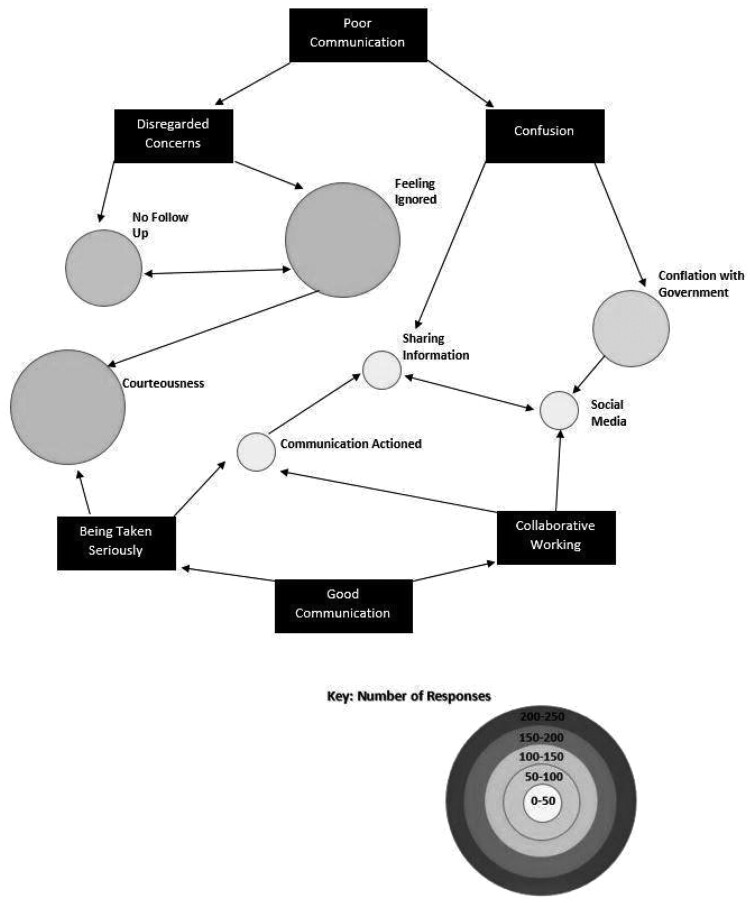
Concept map of free-text responses—communication.

**Figure 2:**
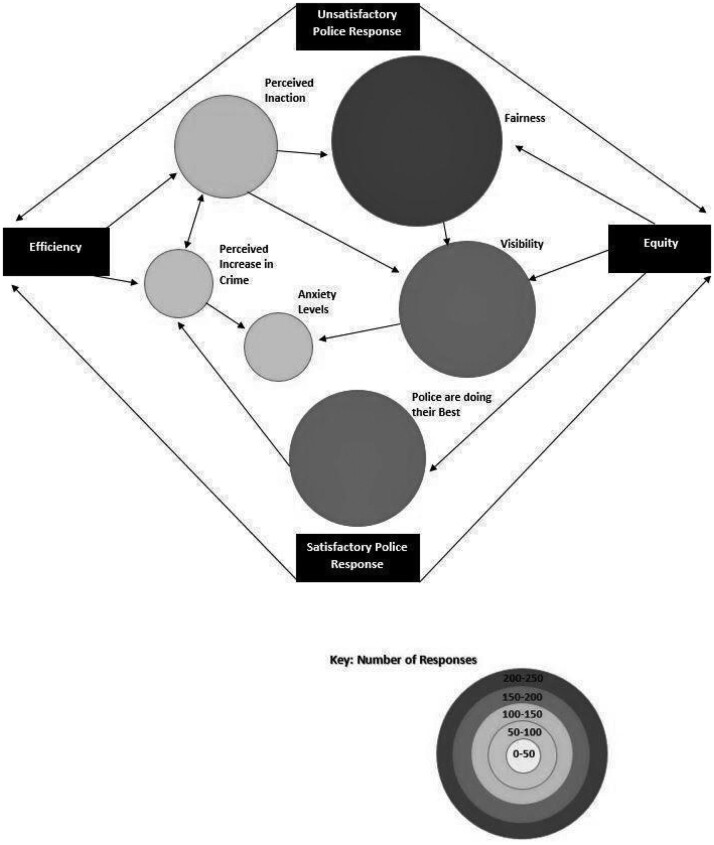
Concept map of free-text responses—police response.

This study has sought—through content analysis—to evaluate the ‘sentiment’ expressed by respondents because their actual knowledge of the police response is largely anecdotal and intensified by media hyperbole (West, 2021). In the first instance, negative and positive comments were identified and then separated from one another. They were then thematically analysed and ordered in terms of the comments content. Responses relating to ‘Communication’ and their opinions on the ‘Police Response’ are the most common themes which were then re-coded into sub-themes that better described both their sentiment and content, with the levels of ‘abstraction’ from the initial themes acting as a mode of categorization (Erlingsson and Brysiewicz, 2017, p. 94). The number of codes relating to each sub-theme is used to denote a hierarchy of importance; however, there is quite a lot of overlap between sub-themes—where the data apply to more than one code. Although the majority of comments are negative towards the policing of restrictions, it would be wrong to suggest that they were wholly negative in scope. In reality, the comments made exist on a continuum between positive and negative opinions. As such, it would be impossible to quantify the sentiment of public opinion regarding police action during the initial lockdown phase, which is why this study has chosen to adopt a more qualitative, thematic approach to its enquiry—to allow for the ‘subjective interpretation’ of the comments to be born out in the analysis (Braun and Clarke, 2021, p. 40). In order to systematize the relationships between the sub-themes, this study has employed the use of concept maps—visual representations of the coding process—which are a useful tool for researchers to both navigate their data and denote the hierarchy of codes and their interconnections (Xiu Guo, 2019). Figures  1 and 2 show the overarching themes, sub-themes, and their relationships to one another; where the nature of responses overlaps.

The content analysis of the free-text responses has not been accumulated to create a verbatim, fact-based dataset. They have instead been analysed to determine both the relationship between viewpoints and the sentiments that drive them, which has been grounded through the premise of public–police relations and their relationships to levels of compliance and police satisfaction found in the existing literature. In the following analysis section, the complex nature of the participant’s responses, where they diverge and where they intersect, is discussed in more detail.

## Findings and analysis

Figures  1 and 2 form the framework for analysis of the free-text comments. The analysis will work inwards from the key themes in each figure, identifying and analysing how these themes are related and linked to the subthemes. The rectangles denote the overarching themes, and each circle signifies a common subtheme found within the free-text data. The size and shade of each circle signifies how many times this point was made by respondents. The arrows indicate how each theme overlaps—where the points made move into related contexts. The figures also work across a continuum of both negative and positive comments, however, these are not always mutually exclusive and therefore each circle is an amalgamation of both sentiments. Detailed descriptions of the number of free-text comments within each circle are outlined in Appendix 1 and are not included within the text to avoid disturbing the flow of the argument.

An important consideration in analysing these themes is that the issues raised in the complexities and challenges presented to the police when engaging with the public (i.e. weighing the logistical realities of their duty of care to ensure public safety against notions of ‘public trust and confidence’) are of course not novel to the Covid-19 pandemic (Laufs and Waseem, 2020). However, balancing the reality of policing a rapidly changing socio-political environment with the expectations of the public is particularly difficult to manage during a national emergency that is also global in scope (Bonkiewicz and Ruback, 2010, pp. 373–374). The public’s ‘risk perception’ of such emergencies, in this case being the public consensus on how dangerous they believe the pandemic is, dictates the nature of communication with the police, as well as the public’s perception of the police’s ability to ensure public safety (Bonkiewicz and Ruback, 2012, p. 140). The comments may reflect issues not confined to the pandemic itself, but issues which the pandemic has amplified for respondents.

### Communication

The overriding theme in Figure  1 is Communication. ‘Poor Communication’, by far the most discussed aspect of communication, is linked to two clear subthemes, ‘Disregarded Concern’ and ‘Confusion’. Disregarded concerns relate to public perceptions that police are actively ignoring the reporting of what they regard as legitimate policing issues: ‘They are absent in many cases of restrictions not being followed and hard to contact.’ This includes behaviour that is ‘normal’ outside of the pandemic such as anti-social behaviour, domestic disturbances, and drug taking and dealing, with ‘teenagers and young adults’ often blamed for ‘forming large groups, taking drugs, intimidating others, stealing from local businesses and vandalizing property.’

There is evidence from the free-text comments that the pandemic has made such activities more visible or increased their frequency with a large number of respondents highlighting ‘low level disorder’ such as social gatherings (107 responses) and anti-social behaviour (51 of the 107 responses). Perceptions of an increase in ‘low level disorder’—that is an increase in anti-social behaviour and public disturbance offences—affects satisfaction with local police teams’ more than sporadic instances of serious crime (Myhill and Bradford, 2012, p. 399). Likewise, the feeling of being ignored was often compounded by the perception that police did not follow-up such complaints. For example, one respondent reported that the police ‘completely failed to respond to reports of large gatherings’, while another perceives that the police are ‘actively avoiding doing anything.’ There was also a common consensus that reporting issues via ringing 101 ‘was hopeless’, with several respondents (28 responses) reporting issues when making contact this way: ‘they even forgot to take my name or give me a reference number on 101.’ Together, these subthemes suggest a relationship between the public feeling disregarded and a perception of a lack of effective policing.

The respondents expressing this sentiment often attempt to rationalize why the police are disregarding their concerns. Some were supportive of the police, suggesting that the guidance on restrictions available to them was not clear and difficult to enforce. Others were less supportive and focused on a perceived unwillingness of the police to put themselves in harm’s way, where they are ‘not prepared to come out and sort the situation as they were concerned for their safety, when they’re there to keep [the public] safe.’

Another subtheme of poor communication was Confusion, which related to issues of policing restrictions as well as the boundaries between the police and other institutions, such as the national government. For the public and police alike, the question of what exactly the ‘rules and expectations’ are when applied at the individual level is a difficult one to answer with any form of consensus (Farrow, 2020). A common complaint made by respondents related to their frustration with the constant ‘tinkering with the rules’ central government appeared to be making, when they would prefer for them to have been made ‘legal requirements rather [than] woolly suggestions.’ This suggests that respondents had little faith in the public’s willingness to voluntary comply with restrictions based on a lack of knowledge on how to do so, where they believe that ‘all rules should have been made compulsory, not a choice.’ For the police, this issue is compounded by the fact that it is they who are tasked with arbitrating lockdown restrictions, with the constant threat of ‘misreading’ the situation impeding their ability to do so through fear of repercussion (Blowe and Lubbers, 2020, p. 19).

It is not unusual for the public to look to policing agencies for direction during instances of widespread confusion, with public expectations placed upon the police to act as ‘the voice of authority, calm, and guidance’ that they need to navigate through any event that causes sustained public distress (Brito *et al.*, 2009, p. 1). What is different about the Covid-19 landscape is the level of overlap in provision between the police and partner agencies—such as the NHS, local, and national government and in some instances, the military, of which there were calls from a small section of respondents (17 responses) for more involvement in terms of policing restrictions: ‘we could have very easily had help from the military in enforcing the new regulations, but why would common sense come into this?’ To be precise, the pace at which legal apparatus has changed to accommodate the nature of the pandemic, as well as the almost constant fluctuations in pandemic-related restrictions, has distorted the perception of boundaries between government and its partner institutions, the police included (Jennings and Perez, 2020). This general confusion is not only experienced by the public—the police have also struggled to determine their role in enforcing lockdown restrictions (Jones, 2020).

Comments (63 responses) indicated respondent’s confused Government guidance with their perception of policing in their local area—where the police are seen as an extension of the State apparatus involved with decision-making. It is interesting to note that the police were not viewed negatively within these comments, but rather as passive agencies deserving of sympathy, where the ‘vague government guidance made it almost impossible for the police to enforce the rules.’ This confusion between the application of the law and Government guidance has a potentially wider impact on ‘voluntary compliance’ as an essential aspect of the ability of the police to manage lockdown restrictions (Grace, 2021, p. 3).

It would be inaccurate, however, to suggest that the fragmentation of the boundaries between the police as a service and the wider State institutional landscape is novel to the Covid-19 landscape. Indeed, many UK police officers have historically complained that their public engagement activities are better suited to other local authority agencies such as social services, with conflation with government policy also commonly reported by officers when engaging with the public (Lane, 2019). What is different is the pace at which these boundary shifts are taking place, compounded by the nature of a perceived incursion upon the freedoms of society in the public consciousness. In juxtaposition, there has been an ‘increased public appetite’ for stricter policing measures, leading to some organizations within the wider State apparatus—such as individual schools—acting independently of the government and imposing school closures because of perceived localized threats (Stott *et al.*, 2020, p. 575).

Understandably, this disparity within the social consciousness—one that shifts daily dependent on changes in government measures and the reaction to these from partner local authority agencies—is difficult for the police to accommodate at the localized level (Reicher and Stott, 2020).

With respect to ‘Good Communication’, key subthemes are ‘Being Taken Seriously’ and ‘Collaborative Working’. Being taken seriously is a prominent concern for the public and is expressed in the comments via two additional subthemes: ‘Courteousness’ and ‘Communication Actioned’ or where the police have provided evidence they have acted on reported public concerns. Comments relating to the professional manner of the police are important for the perception of courteousness, just as the opposite behaviour is associated with poor communication from the police. The perception of a mutual respect in such exchanges, where respondents felt ‘listened to, treated with respect and thanked for [their] assistance’ was key. This positive perception was enhanced where there was overlap with evidence of their communication being actioned, where the respondents report that they were ‘phoned back and followed up’ when they have contacted the police regarding breaches of lockdown restrictions, and the police have provided them with information on how they have handled the issue: ‘the police attended swiftly and took action as appropriate. They took my concerns seriously.’

Communication actioned also overlapped with the second subtheme of good communication: collaborative working. In particular, where there was visible policing, respondents viewed this as evidence of effective public–police collaboration. To be more precise, respondent’s reported a heightened sense of ‘community focused policing’ as a result of the police increasing ‘their patrols of local areas’ due to the pandemic, which has forced them to be ‘more visible’ than they would usually be. Visibility was also an important aspect in the positive response to ‘sharing information and social media’, another component of collaborative working. Several responses (17) highlight that their local police teams *‘*have been more prominent on [social media] with details of where they have patrolled and what they have encountered’ which has been ‘very reassuring.’ Equally, respondents report that social media has allowed them to see the ‘human and vulnerable side’ of policing, which has helped ‘change police perception’ for the better.

The subtheme of social media, like courteousness, does however, also illustrate how public response can differ depending on which route their perception follows: ‘poor’ or ‘good’ communication. Some respondents reported that the police seem to be ‘more interested in posting online’ than dealing with the ‘difficult things going on [like] social distancing and people partying on the beach until 3 am.’ Equally, social media posts have left the police open for criticism on how they were dealing with the pandemic, with members of public reporting they had ‘to correct Facebook updates on what the government guidelines actually are’ which has added to the perception that the pandemic has been poorly managed, where it ‘seems like no one has a clue.’

The advent of social media platforms has made the mechanics of policing, both in the national and localized context, more visible to the communities it is designed to serve (Lee and McGovern, 2014, p. 114). However, this can have both positive and negative effects on the ability of the police to safeguard the populations under their care. In one instance, social media allows the police to communicate the reasoning behind their policing techniques, as well as providing a platform on which to study the behaviours of its targeted communities via engagement—allowing the police to tailor their response to emergent regional shifts in compliance (Wood, 2020, p. 42). Equally, social media platforms allow disinformation to be disseminated relating to policing, especially surrounding events of cultural and social significance such as the pandemic, which require large-scale police intervention (Dekker *et al.*, 2020, p. 5). It is also important to note that the data available to this study are not extensive enough to formulate a firm hypothesis on the information sources used by respondents in order to conclude how interconnected social media activity is to the wider themes.

### Police response

Figure  2 outlines the second key theme that emerged from the analysis of the free-text, the Police Response. As with the communication theme, most of the responses tend to be more negative in scope—‘Unsatisfactory Police Response’*—*which are double that of Satisfactory Police Response. The disparity in responses can partly be explained by the nature of open-ended survey questions, they are most often negative in scope because respondents are more likely to complete them if they have scored the rest of the survey negatively—whereas those who have scored them positively are less likely to contribute free-text responses (Zhou *et al.*, 2017, p. 1279). However, it would be wrong to suggest that the data elicit clear boundaries between negative and positive responses—there are many instances of overlap between both sentiments, with 90 comments made that acknowledge that ‘it was not possible for the police to attend all of the reports that were made, but that was due to their currently reduced numbers and higher demand.’

The two subthemes are mediated by two common subthemes, ‘Efficiency’ of police services and ‘Equity’ of the application of policing powers. The mediation of police performance through these two subthemes corresponds with perceptions of pre-pandemic policing. With respect to efficiency, the criticism often leveled at the police is ‘ambiguous’ in scope, which provides its own challenges; it is impossible for the police to manage their performance in line with public expectations that have little definable contexts beyond perceived discontent (Asif *et al.*, 2018). Many of the criticisms made about the police’s purported efficiency related to a lack of knowledge of the police’s approach, where the police’s actions seemed ‘ad hoc, random and arbitrary.’

It is equally important to note that ‘socioeconomic, demographic, and institutional factors’ such as those wrought by the pandemic have been proven to hinder police effectiveness (Alda *et al.*, 2020, p. 1220). Likewise, the debates surrounding police Efficiency are also often followed by notions of Equity in terms of how the law is applied across different demographics within the population. The perception of the Efficiency of the mechanics of ‘procedural justice’—in this instance the public’s conceptions of the fairness and the transparency of the police in applying the law—often dictate individuals’ overall perceptions of the police and their willingness to both engage and comply with police instruction (Boateng, 2020, p. 986). In juxtaposition to the comments that acknowledge the added pressure put upon the police during the pandemic, some respondents report losing ‘complete faith in the police to police anything’ because they believe that the police are effectively ‘picking and choosing what they enforce’ because the pandemic has given them an ‘excuse to be busy.’

An important link exists between Efficiency and ‘Fairness’. Balancing the usage of effective crime prevention techniques with public opinion on what is fair policing—to increase legitimacy and by proxy levels of compliance—is often a reductive exercise for police forces (Weisburd, 2016, p. 664). The advent of the pandemic has appeared to exacerbate this issue, creating an ‘us and them’ mentality that not only relates to the public’s perceptions of their relationship with the police, but also between the public themselves (Reicher and Stott, 2020, p. 570).

A sense of Fairness was a common sentiment expressed by respondents with an overwhelming majority believing that the lockdown restrictions were not being applied fairly across the population, where the ‘flouting of guidelines has predominantly been ignored by police.’ Sentiments of unfairness were often associated with perceived leniency of policing—focusing on social gatherings—with young people most associated with such gatherings. Respondents made comments such as: ‘more policing less posturing’, ‘too lenient’ and that the police were ‘too worried about negative backlash’ to fully enforce the lockdown rules to compound their points. The common narrative found throughout was that the police were not actively seen policing the lockdown restrictions, linking fairness to both the ‘Visibility’ and ‘Perceived Inaction’ subthemes. This process seems to be affecting the potential compliance of respondents moving forward: ‘I will only comply with what I agree with if there’s another lockdown. There’s no consequences for anyone who doesn’t.’

The link between fairness and the perceived inaction of police can be seen as having two components; the relationship between ‘procedural fairness’—that is to say, how fairly the law is applied to the individual—and ‘distributive fairness’—how evenly the law is applied across the population (Pehrson *et al.*, 2017, pp. 1–2). It is possible to speculate that the Covid-19 pandemic has exacerbated this debate between the two components in the public consciousness. Indeed, there is considerable evidence in the free-text responses of an appetite for more stringent policing—where the police have been perceived as being ‘too lenient’ in their application. This overlaps with the themes of perceived police inaction and a perception of increased levels of crime despite the admittedly sparse evidence currently pointing to a drop in criminal activity as a whole in the wider global context (Ashby, 2020, pp. 15–16).

‘Anxiety’ was also a key concern, linking to perception of an increase in crime and to police visibility, which often acts as a predictor for this subtheme. Examples of emotive language used by respondents such as ‘live in fear’, ‘do not feel safe’, and ‘terrified’ are common. The effects of police visibility on public confidence levels evidently predate the advent of pandemic. Regular foot patrols within target neighbourhoods have been proven to improve feelings of ‘safety’ within surveyed populations (Borovec *et al.*, 2019). It also seems that public satisfaction levels in regard to procedural justice is proportional to both visibility and street level interaction with police officers (Bolger *et al.*, 2021). It was the absence of the visible presence of police that form the basis of most comments linking visibility and anxiety, as one respondent explains: ‘visibility leads to feelings of security.’

‘Anxiety’ can play a significant role in levels of compliance. In the context of the pandemic, if members of the public are sufficiently worried about the risk of infection, they are much more likely to comply with police instruction through the usage of ‘functional fear’ (Harper, 2020, p. 3). Conversely, if left unchecked, individuals and groups who are worried about the pandemic and how it is being managed could exhibit ‘anxiety-avoidance’ behaviours, which can decrease levels of trust in authority and institutions (Wolf *et al.*, 2020).

Moving through the Satisfactory Police Response theme, the same subthemes often emerge, although articulated differently. Fairness, for example, was expressed through comments of gratitude and of professionalism in responses. There was also an appreciation of a perceived focus on more ‘community focused policing’*—*where policing is primarily enacted ‘through patrol activity’—which respondents believe allows the police to respond ‘swiftly to all matters’ needing their attention. The appreciation of a focus on community-based policing is not necessarily related to the lockdown restrictions; community centred approaches have been proven to increase levels of public perceptions of their own ‘safety’, which equally increases levels of compliance and public satisfaction (Shearing, 2016, p. 84). The uncertainty created by the pandemic is likely to have increased the usage of ‘functional fear’—where anxiety surrounding possible infection has promoted a compliant response within the public domain (Solymosi *et al.*, 2020).

Although it is difficult to qualify whether the pandemic has created an increased desire for more visible policing within the public sentiments of the respondents, it is possible to speculate it has intensified this issue, where respondents generally report ‘seeing [the police] around a lot more during the pandemic’ but then going on to compare this to their previous experiences, where they ‘were lucky if we saw them at all.’ The desire to see more police on the streets could be linked to an increased need for clarification on what the restrictions mean in real terms, as one respondent proclaims: ‘I think the restrictions have been virtually impossible to follow, obey and police.’ In short, the lack of ‘previous experience’ with anything resembling lockdown restrictions encourages people to garner access to figures of authority in order to confirm that they have interpreted the guidance correctly (Alcadipani *et al.*, 2020).

The most prominent sub-themes within Satisfactory Police Response were that the ‘Police are doing their Best’. Responses show empathy towards the police having been given a challenging task in exceptional circumstances, where they are ‘at the sharp end of ever changing policy’ that makes it ‘impossible for the police to enforce the current regulations.’ This sentiment was often coupled with a recognition that police were under-resourced, where they are ‘understaffed and overworked’ which makes their job much harder, however they are ‘doing the best they can with limited resources.’ The fact that many respondents showed support towards the police is not necessarily remarkable to the pandemic; public dissatisfaction levels with the police are often over-inflated by media outlets (Moule, 2020, p. 2). It is however possible to speculate that the pandemic has brought this reality to the forefront of respondents’ minds, where they realise that ‘it is a lot to ask an already stretched police force to carry out [the] extra demanding duties’ that the lockdown burdens them with.

## Conclusion

Through identification and classification of the themes from the free-text comments, it is possible to draw a picture of the subthemes and their inter-relationships. From this web, two central themes emerge, ‘satisfactory’ and ‘unsatisfactory’ communication and ‘satisfactory’ and ‘unsatisfactory’ policing. Given that respondents tend to provide unfavourable rather than favourable comments when given the opportunity to feedback in a survey, the emergence of ‘unsatisfactory’ and ‘satisfactory’ strands to these key themes might appear adverse to the police. Central to perceptions of ‘unsatisfactory’ policing is the opinion that public concerns are being disregarded, coupled with a sense of confusion over the role of the police. For ‘satisfactory’ policing, being taken seriously and the development of community focused policing were key positive subthemes.

This dichotomy in public opinion provides the police with several potential policy implications moving forward. To be more precise, overlap between Poor Communication and Good Communication occurs across two main sub-themes: Courteousness and Sharing Information. Courteousness appears to be particularly important to respondents. Although the majority of comments relating to this sub-theme are negative about the police’s ability to communicate with the public on terms they find courteous, when the interaction has been in line with respondent expectations, praise is forthcoming for both the individual officers and the wider police force.

The police’s ability to share information was also an area where both ‘good’ and ‘poor’ perceptions of communication intersect. Although the majority of responses were negative about sharing information, when information is received by respondents they appear satisfied by its content. Providing more avenues for communication, particularly using the medium of social media, is a possible way for the police to increase their engagement with the public moving forward during the pandemic, and beyond. If used effectively, this approach could stem the prevalence of ‘dysfunctional fear’ within the public consciousness, where the pandemic has caused a fear-based response based upon a lack of knowledge on how the police are managing both pandemic-related restrictions and instances of general criminality (Solymosi *et al.*, 2020, pp. 2–3).

Many of the responses within the communication theme are pandemic specific; however, there are linkages with the wider discourse surrounding the barriers to effective public–police relations. Although it is not possible to provide a definitive answer to how much the pandemic has influenced this dynamic, it is possible to postulate on how the confusion the pandemic has created within the public consciousness has exacerbated this issue; the pandemic has confused the line between State and its institutions such as the police, which has challenged the public’s prevailing understanding of who is responsible for their ‘collective security’ (Stott *et al.*, 2020, p. 567).

Confusion over the role of the police also closely relates to the public’s opinions on the effectiveness of the police response. Visibility, that is to say, visual evidence of the police on the beat managing the public, is mostly used as a yardstick in terms of the public’s assessment of police efficiency. This sentiment is often coupled with a sense of fairness—where they perceive that their adherence to the rules is being undermined by the police choosing to not enforce restrictions on those that appear to be breaking them. There is substantial evidence that this is not a novel observation of public expectations in terms of policing (Bowling, 2007; Asif *et al.*, 2018; Boateng, 2020). However, the number of responses that directly reference social gatherings would suggest that this particular debate has been heightened by the lockdown. The comments relating to fairness also suggest that an ‘other’ is being constructed within the public consciousness—where a ‘descriptive norm’ is being established to define those who break restrictions (Lois and Wessa, 2021, p. 21). This finding has influenced the focus of a forthcoming paper from this research project (Inkpen *et al.*, in preparation), where qualitative interviews have taken place with participants of the survey to identify how and where this othering is taking place.

Although there has been more negative commentary aimed at the police in terms of their management of the restrictions, there are many free-text comments that pertain sympathy for the police and the ‘impossible job’ they have been given. Moving forward, it appears that the police will need to strengthen their legitimacy in the post-pandemic world, however there is a clear opportunity to increase this through more ‘face to face’ interactions with the public; the increased visibility during the initial lockdown period was warmly received by many respondents (Myhill and Bradford, 2012, p. 398). Although there are many practicable issues that hamper the police’s ability to increase visibility—mostly attributed to a lack of police numbers—the public are aware of the pressures put upon the police and would welcome more opportunities to work with them in a more collaborative capacity (Murphy, 2017).

In sum, notions of fairness and a want for more police visibility were key to navigating public opinions of satisfactory and unsatisfactory policing, although interpreted in both negative and positive ways. These themes seem to reflect or rather amplify, key perceptions of the public that existed before the pandemic crisis. The pandemic and its policing seem to have thrown these concerns into sharper relief. In terms of the Covid-19 response, the police have been presented with a dichotomy of perspectives. On the one hand, there was the recurring subtheme that illustrated empathy for the police in regard to the difficulty of their task; the perception that, given the lack of resources, the confused messaging and the nature of lockdown restrictions, the police were ‘doing their best’ given the circumstances.

In contrast, further questions have been raised about how effective the policing by consent model has been during lockdown periods; the pandemic has significantly shifted the balance of public opinion towards a desire for a more stringent application of the law. The possible consequences of the long-term effects of this shift are unclear, both in terms of moving forward with the Covid-19 response, and potentially into the post-pandemic world. Regardless, the police will have to continue to navigate the normative discourse generated within the public sphere with due care and diligence if it wishes to preserve the policing by consent model in its current form.

## Appendix

**Table A1:** Number of free-text responses per code

Figure 1: Communication		Figure 2: Police response	
Code	Count	Code	Count
Feeling ignored	121	Fairness	232
Courteousness	115	Visibility	179
Conflation with government	63	Police are doing their best	168
No follow up	58	Perceived inaction	150
Communication actioned	36	Anxiety levels	81
Sharing information	31	Perceived increase in crime	70
Social media	27		
